# Association Between Food Distress and Smoking Among Racially and Ethnically Diverse Adults, Schenectady, New York, 2013–2014

**DOI:** 10.5888/pcd14.160548

**Published:** 2017-08-24

**Authors:** Akiko S. Hosler, Isaac H. Michaels

**Affiliations:** 1 Department of Epidemiology and Biostatistics, University at Albany School of Public Health, Rensselaer, New York

## Abstract

**Introduction:**

Smoking and poor nutrition are 2 leading preventable causes of death. This study investigated associations between smoking and indicators of individual- and neighborhood-level food distress among racially and ethnically diverse urban adults.

**Methods:**

We analyzed data from a health interview survey and a food environment assessment collected in 2013 and 2014 in Schenectady, New York. We constructed logistic regression models for current smoking with 6 indicators of food distress as exposure variables and sociodemographic characteristics, depression, anxiety, perceived stress, alcohol binge drinking, and disability as covariates.

**Results:**

The analytic sample consisted of 1,917 adults; 59.4% were female, more than half were racial/ethnic minorities (26.2% non-Hispanic black, 10.3% Hispanic, 10.9% Guyanese, 4.0% multiracial and other), and 37.1% were current smokers. All indicators of food distress remained in the parsimonious final model: consuming 0 or 1 serving of fruits and vegetables daily more than doubled the odds of smoking, compared with consuming 5 or more servings (odds ratio [OR], 2.05). Food insecurity (OR, 1.77), receiving Supplemental Nutrition Assistance Program benefits (OR, 1.79), using a food pantry (OR, 1.41), living in a neighborhood with low access to healthy food (OR, 1.40), and shopping for food often at a store with limited healthy food choices (OR, 1.38) were also associated with significantly higher odds of smoking.

**Conclusion:**

Recognizing that smoking and food distress are independently associated would lead to innovative public health intervention strategies. We suggest stronger collaboration between tobacco and nutrition public health professionals to synergistically reduce tobacco use and improve nutrition behavior and food environments in communities.

**MEDSCAPE CME**

Medscape, LLC, is pleased to provide online continuing medical education (CME) for this journal article, allowing clinicians the opportunity to earn CME credit.

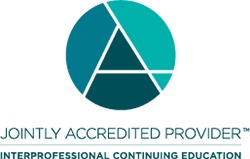
In support of improving patient care, this activity has been planned and implemented by Medscape, LLC, and *Preventing Chronic Disease*. Medscape, LLC, is jointly accredited by the Accreditation Council for Continuing Medical Education (ACCME), the Accreditation Council for Pharmacy Education (ACPE), and the American Nurses Credentialing Center (ANCC), to provide continuing education for the healthcare team.

Medscape, LLC, designates this Journal-based CME activity for a maximum of 1.00 *AMA PRA Category 1 Credit(s)™*. Physicians should claim only the credit commensurate with the extent of their participation in the activity.

All other clinicians completing this activity will be issued a certificate of participation. To participate in this journal CME activity: (1) review the learning objectives and author disclosures; (2) study the education content; (3) take the post-test with a 75% minimum passing score and complete the evaluation at http://www.medscape.org/journal/pcd; (4) view/print certificate.

**Release date: August 24, 2017; Expiration date: August 24, 2018**

**Learning Objectives**

Upon completion of this activity, participants will be able to:

1. Assess risk factors for smoking and trends in the epidemiology of smoking
2. Analyze risk factors for smoking in the current study
3. Distinguish the most salient elements of food insecurity associated with smoking in the current study
4. Evaluate different retail locations in terms of their display of tobacco advertising

**EDITOR**

Ellen Taratus, MS

Editor, *Preventing Chronic Disease*

Disclosure: Ellen Taratus, MS, has disclosed no relevant financial relationships.

**CME AUTHOR**

Charles P. Vega, MD

Health Sciences Clinical Professor, University of California, Irvine, Department of Family Medicine; Associate Dean for Diversity and Inclusion, University of California, Irvine, School of Medicine, Irvine, California

Disclosure: Charles P. Vega, MD, has disclosed the following relevant financial relationships:

Served as an advisor or consultant for: McNeil Consumer Healthcare

Served as a speaker or a member of a speakers bureau for: Shire Pharmaceuticals

**AUTHORS**

Akiko S. Hosler, PhD

Department of Epidemiology and Biostatistics, University at Albany School of Public Health, Rensselaer, New York

Disclosure: Akiko S. Hosler, PhD, has disclosed no relevant financial relationships.

Isaac H. Michaels, MPH

Department of Epidemiology and Biostatistics, University at Albany School of Public Health, Rensselaer, New York

Disclosure: Isaac H. Michaels, MPH, has disclosed no relevant financial relationships.

## Introduction

Smoking is the leading preventable cause of death in the United States (1). Although smoking prevalence among adults declined from nearly 45% in the 1960s to 17% in 2014, the prevalence of smoking and its adverse health consequences is still disproportionately high among certain segments of society (2). For example, the prevalence of smoking is higher among American Indians than among other racial/ethnic groups (2), and people with low income or low educational attainment are more likely to smoke than people with higher socioeconomic status (2,3). Several modifiable behavioral factors and health conditions are associated with smoking, including alcohol binge drinking (4–6), stress (5–8), depression (6,9,10), anxiety disorders (9,11), mental illness (12), and disability (2,13).

Poor nutrition is another major preventable cause of death (1), but its association with smoking has not been extensively investigated. Smokers are less likely than nonsmokers to consume fruits and vegetables and thus have less intake of folate, vitamin C, and fiber (14,15). Food insecurity, a condition in which an individual or household perceives a lack of sufficient resources to obtain safe and nutritionally adequate foods, is also independently associated with smoking (16–19). Research on the association between smoking and environmental indicators of food distress (a comprehensive concept of food and nutrition inadequacy, ranging from poor dietary behavior and food insecurity to lack of access to healthful food and insufficient or ineffective food and nutrition policy) is sparse. Living in a neighborhood with little availability of healthy foods and shopping for food in stores that offer few healthy food choices are linked with poor dietary behavior (20–22), and these factors could be directly associated with smoking. Identifying new modifiable risk factors or health consequences of smoking can lead to innovative public health strategies for smoking prevention, cessation, and policy development. The objective of this study was to investigate whether current smoking has independent associations with individual- or neighborhood-level indicators of food distress in a sample of racially and ethnically diverse adults residing in an urban community.

## Methods

This study took place in Schenectady, New York. This city of approximately 66,000 residents is designated as a priority community for health improvement by a local public health coalition because of the high level of chronic disease risks, including a high prevalence of smoking among adults. Our university-based research team has been a core member of this coalition since its inception in 2012. The institutional review boards of the University at Albany and Ellis Hospital reviewed and approved the human subject protection protocol of this research.

**Health interview survey.** We conducted a door-to-door cross-sectional health interview survey from February to May 2013. Schenectady residents aged 18 years or older who were able to understand informed consent written in English or Spanish were invited to complete the survey. A team of trained survey administrators, many of whom were community health workers, canvassed all 10 administratively defined Schenectady neighborhoods. Approximately 1,400 private homes, including units in senior apartment buildings, municipal housing, and commercial apartment complexes were visited. One eligible adult was interviewed per household. Additionally, the team visited 36 community venues and events to interview eligible individuals who were hard to reach through home visits. We set a target sample size (quota) for each neighborhood, so that the total sample size would have proportionately representative neighborhood subsamples. An incentive (a gift card worth $10) was given to each participant at completion of the interview.

**Food environment assessment.** From May through August 2014, we conducted a food environment assessment in the city of Schenectady and its walkable adjacent area up to 1 street-network mile beyond the city’s boundaries. We obtained 6 government administrative lists of retailers to identify locations of all food stores in the study area. These were a list of inspected food stores from the New York State Department of Agriculture and Markets, a list of authorized retailers for the Supplemental Nutrition Assistance Program (SNAP) from the US Department of Agriculture, a list of registered cigarette retailers from the New York State Department of Taxation and Finance, a list of authorized lottery retailers from the New York State Lottery, a list of off-premises liquor licenses from the New York State Liquor Authority, and a list of authorized retailers for the Special Supplemental Nutrition Program for Women, Infants, and Children (WIC) from the New York State Department of Health. A food store was defined as a retail outlet that sold milk, loaves of bread, or fruits or vegetables that were fresh, frozen, or canned. A team of trained research personnel canvassed the city to verify stores’ eligibility and find stores not on the lists. We conducted the in-store assessment using the Food Retail Outlet Survey Tool, a paper tool that has high interrater reliability (κ ≥0.85) (23). We also assessed the presence of types of fresh fruits and vegetables, the presence of tobacco products, and the presence of tobacco advertising. All stores granted permission to conduct the assessment.

### Measures

**Current smoking.** The outcome variable of this study was current smoking. Respondents who indicated they had smoked at least 100 cigarettes in their lifetime and smoked cigarettes every day or some days at the time of survey were considered current smokers.

**Food distress.** Six variables were selected to measure various aspects of food distress: 1) fruit and vegetable consumption, 2) food insecurity, 3) use of a food pantry, 4) participation in SNAP, 5) neighborhood access to healthy food, and 6) frequency of shopping for food at a corner store, dollar store, or drug store. Fruit and vegetable consumption was measured as the total number of servings on an average day. Food insecurity was defined as not having had enough food to eat at home often or sometimes in the previous 12 months. This measure was created for our health interview survey to assess food insecurity in the community survey setting. A household’s use of a food pantry and participation in SNAP were assessed by yes/no questions. For measures of the food environment, we analyzed data from the food environment assessment. Schenectady’s 10 neighborhoods were grouped into 2 categories of access to healthy food: low and moderate. We defined a neighborhood having low access to healthy food as 1) being more than 1 street-network mile from the geometric center of inhabited areas of the neighborhood to the nearest supermarket (24) and 2) having fewer than 5.0 stores per 10,000 population density that carry at least 2 types of fresh fruits (excluding lemons and limes) and 2 types of dark-colored fresh vegetables (25). We defined a neighborhood having moderate access to healthy food as 1) being within 1 street-network mile from the geometric center of inhabited areas of the neighborhood to the nearest supermarket and 2) having fewer than 10.0 stores per 10,000 population density that carry at least 2 types of fresh fruits (excluding lemons and limes) and 2 types of dark-colored fresh vegetables. The food environment assessment data indicated that corner (convenience) stores, dollar stores, and drug stores were types of stores that were least likely to have adequate types of fruits and vegetables and other healthy foods. On the basis of this knowledge, we consolidated self-ratings of shopping for food “often” at any of these types of stores into a variable to indicate shopping at a store with limited healthy food choices.

**Sociodemographic characteristics.** Sociodemographic variables were age, sex, race/ethnicity, educational attainment, and household income. Guyanese participants were grouped as a distinctive racial/ethnic category; they are English-speaking South American people of Asian Indian descent (Indo-Guyanese). Because nearly one-third of respondents did not report their income, we created a category for “income not reported.”

**Health conditions and behavioral factors. **We selected 5 health conditions and behavioral factors that are associated with smoking. Anxiety disorder was defined as an affirmative response to the question “Have you ever been told by a health professional that you have/had an anxiety problem?” and reporting of any one of the following: 1) is currently taking medication or receiving treatment for anxiety, 2) saw a health care professional for a routine checkup related to anxiety in the previous 12 months, 3) received care at an emergency department for anxiety in the previous 12 months, or 4) was hospitalized because of anxiety in the previous 12 months. Likewise, depression was defined as the combination of ever being told by a health care professional that the respondent has or had depression and reporting of current treatment or medication use for depression or any depression-related routine or emergent health care use in the previous 12 months. Alcohol binge drinking was defined as having had 5 or more alcoholic drinks on one occasion in the previous 30 days. The 10-item Perceived Stress Scale (PSS-10) was used for defining recent stress (26); each item is scored on a scale of 0 to 4 (for a total possible score ranging from 0 to 40). We defined high stress as a PSS-10 score of 20 or greater. Disability was defined as 1) having any health problem that requires special equipment such as a cane, a wheelchair, a special bed, or a special telephone, 2) reporting disability as a reason for not working, or 3) receiving Social Security Disability benefits and/or Supplemental Security Income disability benefits.

### Data analysis

We calculated frequencies and percentage distribution for all variables and compared the prevalence of current smoking across categories for each variable. We used a Pearson χ^2^ test to evaluate significant differences in smoking prevalence. Complete case analysis was used to handle missing data, and an α of .01 was used to assess significance. We performed multivariable logistic regression analyses to investigate the association between current smoking and indicators of food distress, with sociodemographic, health, and behavioral variables as covariates. We used the backward stepwise technique to remove nonsignificant variables to obtain a parsimonious model. For the selection of variables for removal, we used the probability of the likelihood-ratio statistic based on conditional parameter estimates. We also conducted stratified analyses to confirm the lack of effect modification in the association between food distress indicators and smoking by sociodemographic, health, and behavioral variables. We estimated odds ratios (ORs), 95% confidence intervals (CIs), and *P* values for the final model. We conducted an additional analysis to examine the neighborhood food environment and its relationship with the tobacco environment by comparing the presence of tobacco products, the presence of tobacco advertising, and the availability of fresh fruits and vegetables in various types of food stores. All statistical analyses were performed by using SPSS-PC version 23.0 (IBM Corporation).

## Results

The analytic sample consisted of 1,917 adults; 59.4% of the sample were female, and the mean age was 45.5 years. More than half were racial/ethnic minority respondents, including non-Hispanic black (26.2%), Guyanese (10.9%), Hispanic (10.3%), and multiracial/other race respondents (4.0%). The prevalence of current cigarette smoking was 37.1% for the sample overall, and it varied by subgroup (Table 1). Smoking prevalence differed significantly within categories for all sociodemographic, health, behavioral, and food distress variables. We found a prevalence of 50% or higher among those who reported anxiety disorder, depression, alcohol binge drinking, consuming 0 or 1 serving of fruits and vegetables per day, food insecurity, using a food pantry, and receiving SNAP benefits.

**Table 1:** Sociodemographic and Health-Related Characteristics of Participants in a Health Interview Survey, Schenectady, New York, 2013–2014

Characteristic	No. (%)	Current Cigarette Smokers, %	*P* Value
**Sample total**	1,917 (100.0)	37.1	—
**Sociodemographic Characteristics**			
**Age, y**			
18–34	549 (28.6)	39.5	<.001
35–54	829 (43.2)	45.1	
≥55	539 (28.1)	22.3	
**Sex**			
Female	1,139 (59.4)	34.2	.002
Male	778 (40.6)	41.3	
**Race/ethnicity**			
Non-Hispanic white	933 (48.7)	33.7	<.001
Non-Hispanic black	503 (26.2)	46.3	
Hispanic	197 (10.3)	46.2	
Guyanese	208 (10.9)	22.6	
Other	76 (4.0)	34.2	
**Educational attainment**			
<High school diploma	288 (15.0)	45.8	<.001
High school diploma/GED	1,175 (61.3)	39.3	
≥Some college	454 (23.7)	25.8	
**Annual household income, $**			
<20,000	821 (42.8)	44.7	<.001
20,000–49,999	326 (17.0)	24.8	
≥50,000	149 (7.8)	14.1	
Not reported	621 (32.4)	39.0	
**Health Conditions and Behavioral Factors**			
**Anxiety disorder**			
Yes	282 (14.7)	55.7	<.001
No	1,635 (85.3)	33.9	
**Depression**			
Yes	362 (18.9)	52.8	<.001
No	1,555 (81.1)	33.4	
**Alcohol binge drinking^a^**			
Yes	367 (19.1)	53.7	<.001
No	1,550 (80.9)	33.2	
**Perceived Stress Scale^b^**			
Score ≥20	502 (26.2)	49.2	<.001
Score <20	1,415 (73.8)	32.8	
**Disability**			
Yes	631 (32.9)	44.8	<.001
No	1,286 (67.1)	33.3	
**Food Distress Indicators**			
**Fruit and vegetable consumption per day, servings**			
0 or 1	294 (15.3)	51.7	<.001
2–4	1,028 (53.6)	35.5	
≥5	595 (31.0)	32.6	
**Not enough food to eat at home**			
Often or sometimes	331 (17.3)	57.1	<.001
Rarely or never	1,586 (82.7)	32.9	
**Use a food pantry**			
Yes	421 (22.0)	52.3	<.001
No	1,496 (78.0)	32.8	
**Receive SNAP benefit**			
Yes	789 (41.2)	51.7	<.001
No	1,128 (58.8)	26.9	
**Neighborhood food environment**			
Low access to healthy food	1,124 (58.6)	43.5	<.001
Moderate access to healthy food	793 (41.4)	28.0	
**Shop for food often at a corner/dollar/drug store**			
Yes	812 (42.4)	46.4	<.001
No	1,105 (57.6)	30.2	

Abbreviations: —, not applicable; SNAP, Supplemental Nutrition Assistance Program.

a Defined as having had 5 or more alcoholic drinks on one occasion in the previous 30 days.

b The 10-item Perceived Stress Scale (PSS-10) was used for defining recent stress (26); each item is scored on a scale of 0 to 4 (for a total possible score ranging from 0 to 40). We defined high stress as a PSS-10 score of 20 or greater.

All food distress indicators remained in the parsimonious final model (Table 2). Respondents who consumed 0 or 1 serving of fruits and vegetables per day had significantly higher odds (OR, 2.05) of current smoking compared with those who consumed 5 or more servings of fruits and vegetables. Similarly, we found significantly higher odds of current smoking among respondents who were food insecure (OR, 1.77), used a food pantry (OR 1.41), received SNAP benefits (OR 1.79), resided in a neighborhood with low access to healthy food (OR 1.40), and shopped for food often at a corner store, dollar store, or drug store (OR 1.38), compared with their reference groups. In addition, age younger than 55 years, annual household income less than $20,000, anxiety disorder, and alcohol binge drinking were associated with significantly higher odds of smoking, while female sex and Guyanese race/ethnicity were associated with significantly lower odds of smoking. Educational attainment, depression, perceived stress, and disability were removed from the model because their contributions to the model were not significant. The stratified analyses found no significant effect modification by sociodemographic, health, or behavioral variables. The final model had a Nagelkerke pseudo *R* ^2^ of 0.30.

**Table 2:** Results of a Multivariate Logistic Regression Model For Current Smoking, by Sociodemographic Characteristics, Health-Related Factors, and Food Distress Indicators, Schenectady, New York, 2013–2014^a^

Variable	Odds Ratio (95% Confidence Interval)	*P* Value
**Sociodemographic Characteristics**		
**Age, y**		
18–34	1.87 (1.39–2.50)	<.001
35–54	2.28 (1.73–2.98)	<.001
≥55	1 [Reference]	
**Sex**		
Female	0.63 (0.51–0.78)	<.001
Male	1 [Reference]	
**Race/ethnicity**		
Non-Hispanic white	1 [Reference]	
Non-Hispanic black	0.91 (0.70–1.19)	.50
Hispanic	1.01 (0.71–1.43)	.96
Guyanese	0.33 (0.22–0.50)	<.001
Other	0.77 (0.45–1.30)	.32
**Annual household income, $**		
<20,000	2.29 (1.35–3.89)	.002
20,000–49,999	1.78 (1.03–3.06)	.04
≥50,000	1 [Reference]	
Not reported	1.64 (0.95–2.83)	.08
**Health Conditions and Behavioral Factors**		
**Anxiety disorder**		
Yes	1.71 (1.28–2.29)	<.001
No	1 [Reference]	
**Alcohol binge drinking^b^**		
Yes	2.01 (1.55–2.59)	<.001
No	1 [Reference]	
**Food Distress Indicators**		
**Not enough food to eat at home**		
Often or sometimes	1.77 (1.35–2.33)	<.001
Rarely or never	1 [Reference]	
**Fruit and vegetable consumption per day, servings**		
0 or 1	2.05 (1.49–2.81)	<.001
2–4	1.32 (1.04–1.67)	.02
≥5	1 [Reference]	
**Use a food pantry**		
Yes	1.41 (1.09–1.83)	.009
No	1 [Reference]	
**Receive SNAP benefits**		
Yes	1.79 (1.42–2.26)	<.001
No	1 [Reference]	
**Neighborhood food environment**		
Low access to healthy food	1.40 (1.11–1.77)	.005
Moderate access to healthy food	1 [Reference]	
**Shop for food often at corner/dollar/drug store**		
Yes	1.38 (1.10–1.73)	.005
No	1 [Reference]	

Abbreviations: SNAP, Supplemental Nutrition Assistance Program

a Educational attainment, depression, perceived stress, and disability were removed from the model because their contributions to the model were not significant.

b Defined as having had 5 or more alcoholic drinks on one occasion in the previous 30 days.

Five neighborhoods that had low access to healthy food were clustered in the inner part of the city (Figure). Eight of 9 of the city’s food pantries were in these neighborhoods. Corner stores, dollar stores, and drug stores were numerous, and they were spread fairly evenly across the city and its outer areas; these types of stores were associated with high levels of tobacco products and tobacco advertising (Table 3). All corners stores and drug stores and 62.5% of dollar stores were tobacco retailers, and nearly all of these tobacco retailers had tobacco advertising.

**Figure:**
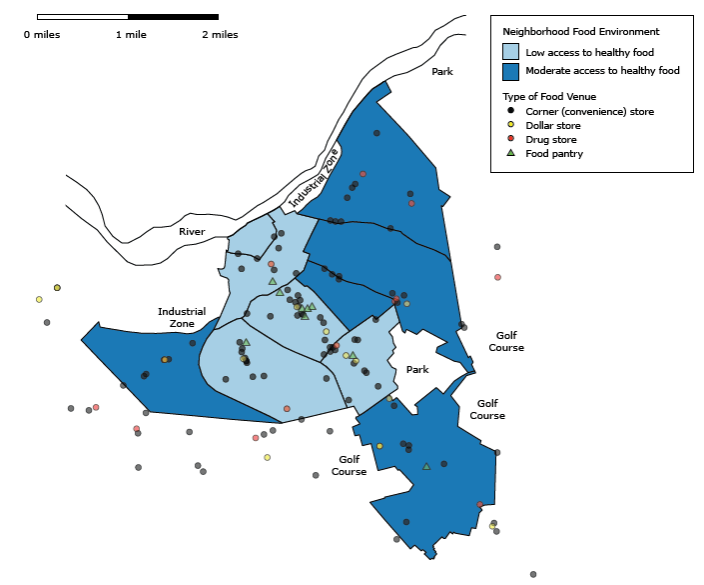
Food environment of Schenectady, New York, 2014. We defined a neighborhood having low access to healthy food as 1) being more than 1 street-network mile from the geometric center of inhabited areas of the neighborhood to the nearest supermarket (24) and 2) having fewer than 5.0 stores per 10,000 population density that carry at least 2 types of fresh fruits (excluding lemons and limes) and 2 types of dark-colored fresh vegetables (25). We defined a neighborhood having moderate access to healthy food as 1) being within 1 street-network mile from the geometric center of inhabited areas of the neighborhood to the nearest supermarket and 2) having fewer than 10.0 stores per 10,000 population density that carry at least 2 types of fresh fruits (excluding lemons and limes) and 2 types of dark-colored fresh vegetables.

**Table 3:** Availability of Fresh Fruits and Vegetables, Tobacco, and Tobacco Advertising by Food Store Type, Schenectady, New York, 2013–2014

Store type	No. of Stores	Sells Fresh Fruits and Vegetables^a^, No. (%)	Sells Tobacco, No. (%)	Has Tobacco Advertising, No. (%)
Corner store	97	8 (8.2)	97 (100.0)	92 (94.8)
Dollar store	16	0	10 (62.5)	10 (62.5)
Drug store	13	0	13 (100.0)	13 (100.0)
Supermarket	12	12 (100.0)	6 (50.0)	2 (16.7)
Food cooperative	1	1 (100.0)	0	0
Small grocery store	12	6 (50.0)	3 (25.0)	3 (25.0)
Miscellaneous store types^b^	12	2 (16.7)	2 (16.7)	2 (16.7)
Total	163	29 (17.8)	131 (80.4)	122 (74.8)

a Sell at least 2 types of fresh fruits (excluding lemons and limes) and 2 types of fresh dark-colored vegetables.

b Includes bakery, meat, beverage, and prepared food stores.

## Discussion

Our study demonstrated that each of the 6 indicators of food distress was significantly associated with current smoking, using a multivariable statistical model where sociodemographic, health, and behavioral variables were covariates. The independent associations between low rates of consumption of fruits and vegetables and smoking (14,15) and between food insecurity and smoking (16–19) have been reported, and our study confirmed these relationships in a sample of racially and ethnically diverse adults in Schenectady, New York. Our study was the first to report that participation in SNAP, use of a food pantry, living in a neighborhood with low access to healthy food, and shopping for food often in a store with limited healthy food choices were also independently associated with smoking.

Research on the mechanism of the linkage between smoking and food distress is limited. A common explanation for a higher occurrence of food insecurity, poor nutrition, and reliance on a nutrition assistance program among smokers than among nonsmokers is the “opportunity cost” argument. New York State has the highest cigarette excise tax in the nation ($4.38 per pack since 2010) (27), and the average cost of a pack of cigarettes in the state is $10.29. The argument is that smokers spend a large portion of their expendable money on cigarettes (up to 24% of income), leaving them less money to spend on food (27,28). Research also points out that smokers tend to have less of an appetite for food than nonsmokers (16), possibly because their palate and hunger-satiety sensation are altered by smoking. Conversely, chronic hunger, imbalanced diet, and economic difficulty in obtaining adequate food can cause stress and anxiety and increase dependence on nicotine. Food-insecure people also smoke to suppress hunger; thus, the relationship is reciprocal (18).

The independent association between living in a neighborhood with low access to healthy food and smoking is an intriguing finding. A poor neighborhood food environment might be an additional source of stress, anxiety, and hunger that would intensify nicotine addiction. It could also be a manifestation of poor dietary behavior and a low demand for healthy food aggregated among residents. In our study, the prevalence of smoking was higher in neighborhoods with low access to healthy food (43.5%) than in neighborhoods with moderate access (28.0%).

The association between smoking and shopping for food often at a corner store, dollar store, or drug store can be partially explained by the in-store tobacco environment. These 3 types of stores not only had the most limited healthy food choices but also had the most pro-tobacco in-store environment, indicated by the high prevalence of tobacco products and tobacco advertising. Point-of-purchase (POP) tobacco advertising, which is currently not regulated by any local law in Schenectady, can increase cravings to smoke, entice impulsive purchases of cigarettes, and create barriers to smoking cessation efforts (29). Although 6 of the 12 supermarkets in our study also sold cigarettes, none displayed cigarettes in a case, and only 2 supermarkets had POP tobacco advertising.

Our study has limitations. We cannot draw conclusions about causality because the study was cross-sectional. The self-reported data on health conditions and behaviors were limited by recall and social desirability biases. Other covariates that may be associated with smoking were not measured in our study. In particular, levels of physical activity may modify the association between food distress and smoking. We did not collect data on the frequency of smoking or the numbers of cigarettes smoked.

Recognizing that both individual- and neighborhood-level indicators of food distress and smoking are independently associated has implications for public health practice. Community nutrition assistance resources, such as food pantries and soup kitchens, SNAP and other adult nutrition assistance programs, and hunger prevention activity sites can be important means to reach smokers, disseminate smoking-related educational materials, and provide referrals to smoking cessation programs. Areas identified as food deserts by the US Department of Agriculture (24) are likely to have a high prevalence of smoking among adults, and this information on food deserts can help target communities for tighter environmental tobacco control and increased community-based smoking cessation efforts. For eliminating pro-tobacco in-store environments, convenience stores, dollar stores, and drug stores should be prioritized for intervention. The ongoing public health campaign to ban tobacco sales in drug stores can be combined with an initiative to increase healthy food choices and nutrition guidance, by demonstrating to the drug store industry that consumers are increasingly interested in healthful products (30).

Improving the nutrition environment while minimizing the pro-tobacco environment in a widespread and sustainable manner calls for policy change. Only a few policy-based interventions exist. The city of Baldwin Park, California, enacted the nation’s first citywide healthy corner store policy in August 2014 (31). This policy incentivizes local small business to increase healthy food selections and imposes tobacco control measures such as eliminating visible tobacco displays from checkout counters, replacing tobacco posters with health education posters, and reducing tobacco exterior signage, although it does not include any tobacco sales restrictions (31). We suggest that the tobacco control community increase collaborative efforts with the nutrition and obesity prevention community by actively participating in the “healthy store” movement to synergistically reduce tobacco use and improve dietary behavior through policy changes.

## Post-Test Information

To obtain credit, you should first read the journal article. After reading the article, you should be able to answer the following, related, multiple-choice questions. To complete the questions (with a minimum 75% passing score) and earn continuing medical education (CME) credit, please go to http://www.medscape.org/journal/pcd. Credit cannot be obtained for tests completed on paper, although you may use the worksheet below to keep a record of your answers.

You must be a registered user on http://www.medscape.org. If you are not registered on http://www.medscape.org, please click on the “Register” link on the right hand side of the website.

Only one answer is correct for each question. Once you successfully answer all post-test questions, you will be able to view and/or print your certificate. For questions regarding this activity, contact the accredited provider, CME@medscape.net. For technical assistance, contact CME@medscape.net. American Medical Association’s Physician’s Recognition Award (AMA PRA) credits are accepted in the US as evidence of participation in CME activities. For further information on this award, please go to https://www.ama-assn.org. The AMA has determined that physicians not licensed in the US who participate in this CME activity are eligible for *AMA PRA Category 1 Credits™*. Through agreements that the AMA has made with agencies in some countries, AMA PRA credit may be acceptable as evidence of participation in CME activities. If you are not licensed in the US, please complete the questions online, print the AMA PRA CME credit certificate, and present it to your national medical association for review.

## Post-Test Questions

### Study Title: Association Between Food Distress and Smoking Among Racially and Ethnically Diverse Adults, Schenectady, New York, 2013–2014

#### CME Questions

1. You are seeing an 18-year-old man who began smoking cigarettes several weeks ago. What should you consider regarding the epidemiology of smoking and associated behaviors as you counsel this patient about his smoking habit?
  - Smoking prevalence among US adults was nearly 40% in 2014
  - There is little difference in the prevalence of smoking in comparing different racial/ethnic groups
  - Low educational attainment and low income are associated with higher rates of smoking
  - Dietary habits are poorly correlated with smoking overall
2. Which one of the following variables was most associated with a significantly higher risk of smoking in the current study?
  - Receiving SNAP benefit
  - Female sex
  - Guyanese ethnicity
  - Age 55 years or older
3. You take a thorough social history on this patient. Which one of the following variables was the only one associated with more than a 2-fold increase in the odds ratio for smoking?
  - Use of a food pantry
  - Shopping for food often at a corner/dollar/drug store
  - Receiving SNAP benefit
  - Consuming 0 to 1 servings of fruits and vegetables per day
4. You talk to the patient about where he and his family shop for food. Which one of the following retail locations was associated with the highest rate of tobacco advertising in the current study?
  - Drug stores
  - Small grocery stores
  - Dollar stores
  - Supermarket
